# Supplementary material for: Cdc6 is sequentially regulated by PP2A-Cdc55, Cdc14, and Sic1 for origin licensing in S. cerevisiae
Source: eLife. 2022 Feb 10;11:e74437. doi: 10.7554/eLife.74437 (PMC8830886; doi:10.7554/eLife.74437)
Supplement: Supplementary file 1. [file elife-74437-supp1.docx]

Supplementary File 1. Strain List

| Strain# | Genotypes | Origin |
| --- | --- | --- |
| JPY003 | *MATa bar1 CDC6-9MYC::TRP1 ADE2 leu2-3 trp1-1 ura3-1 his3-11* | This study |
| JPY009 | *MATa bar1 Δclb2::ADE2 CDC6-9MYC::TRP1 ade2-1 leu2-3 trp1-1 ura3-1 his3-11* | This study |
| JPY011 | *MATa bar1 Δcdc55::KanMX CDC6-9MYC::TRP1 ADE2 leu2-3 trp1-1 ura3-1 his3-11* | This study |
| JPY005 | *MATalpha CDC6-9MYC::TRP1 Δclb5::URA3 ade2-1 leu2-3 trp1-1 ura3-1 his3-11* | This study |
| JPY002 | *MATa bar1 CDC6-9MYC::TRP1 Δrts1::KanMX ADE2 leu2-3 trp1-1 ura3-1 his3-11* | This study |
| JPY029 | *MATa bar 1 CDC6-prA::HIS3 ade2-1 leu2-3 trp1-1 ura3-1 his3-11* | This study |
| JPY030 | *MATa bar 1 CDC6-prA::HIS3 Δclb2::LEU2 ade2-1 leu2-3 trp1-1 ura3-1 his3-11* | This study |
| JPY032 | *MATa bar 1 CDC6-PRA::HIS CDC55-MYC::KanMX ade2-1 leu2-3 trp1-1 ura3-1 his3-11* | This study |
| JPY033 | *MATa bar1 CDC6-PRA::HIS cdc55-101-MYC::KanMX ade2-1 leu2-3 trp1-1 ura3-1 his3-11* | This study |
| JPY183 | *MATalpha whi5::Whi5-mCherry::SpHIS5 ura3::PCDC6-CDC6-yeCitrine::URA3 cdc6::natNT2::GALS-3HA-CDC6 ADE2 leu2-3 trp1-1 ura3-1 his3-11* | from Mart Loog |
| JPY175 | *MATalpha cdc55-101-MYC::KanMX whi5::Whi5-mCherry::SpHIS5 ura3::PCDC6-CDC6-yeCitrine::URA3 cdc6::natNT2::GALS-3HA-CDC6 ADE2 leu2-3 trp1-1 ura3-1 his3-11* | This study |
| JPY174 | *MATalpha Δcdc55::KanMX whi5::Whi5-mCherry::SpHIS5 ura3::PCDC6-CDC6-yeCitrine::URA3 cdc6::natNT2::GALS-3HA-CDC6 ADE2 leu2-3 trp1-1 ura3-1 his3-11* | This study |
| JPY193 | *MATalpha CDC6-GFP::HIS3 ADE2 leu2-3 trp1-1 ura3-1 his3-11* | This study |
| JPY192 | *MATalpha cdc55-101-MYC:KanMX CDC6-GFP::HIS3 ADE2 leu2-3 trp1-1 ura3-1 his3-11* | This study |
| JPY056 | *MATa bar 1 CDC6-T368A-PRA::HIS3 ade2-1 leu2-3 trp1-1 ura3-1 his3-11* | This study |
| JPY057 | *MATa bar 1 CDC6-T368A-PRA::HIS3 Δclb2::LEU2 ade2-1 leu2-3 trp1-1 ura3-1 his3-11* | This study |
| JPY048 | *MATalpha Δclb2::LEU2 ade2-1 leu2-3 trp1-1 ura3-1 his3-11* | This study |
| BCY581 | *MATa bar1 CDC6-T39A-T368A-prA::HIS3 ade2-1 leu2-3 trp1-1 ura3-1 his3-11* | This study |
| JPY123 | *MATa bar 1 URA3::CDC6-T7A-T23A-T39A-S43A-T368A-S372A-13MYC ADE2 leu2-3 trp1-1 his3-11* | This study |
| JPY126 | *MATalpha Δcdc55:KanMX URA3::CDC6-T7A-T23A-T39A-S43A-T368A-S372A-13MYC ade2-1 leu2-3 trp1-1 his3-11* | This study |
| JPY124 | *MATa bar 1 URA3::CDC6-T23A-T39A-S43A-T368A-S372A-13MYC ADE2 leu2-3 trp1-1 his3-11* | This study |
| JPY127 | *MATa bar 1 Δcdc55:KanMX URA3::CDC6-T23A-T39A-S43A-T368A-S372A-13MYC ade2-1 leu2-3 trp1-1 his3-11* | This study |
| JPY087 | *MATalpha URA3::GAL-SIC1-HA CDC6-T39A-T368A-prA::HIS3 Δcdh1::LEU2 ade2-1 leu2-3 trp1-1 his3-11* | This study |
| YDR12 | *MATa cdc15-2 bar1::kanMX pep4::HIS3 ura3::Pgal1,10-CDC6-TAPtcp (URA3) ade2-1 ura3-1 his3-11,15 trp1-1 leu2-3,112 can1-100* | from Dirk Remus |
| JPY069 | *MATalpha URA3::GAL-CDC6-HA ADE2 leu2-3 trp1-1 his3-11* | This study |
| JPY066 | *MATalpha Δclb2::LEU2 URA3::GAL-CDC6-HA ADE2 leu2-3 trp1-1 his3-11* | This study |
| JPY068 | *MATa URA3::GAL-CDC6-T368A-HA ADE2 leu2-3 trp1-1 his3-11* | This study |
| JPY065 | *MATalpha Δclb2::LEU2 URA3::GAL-CDC6-T368A-HA ADE2 leu2-3 trp1-1 his3-11* | This study |
| JPY067 | *MATalpha URA3::GAL-CDC6-T39A-T368A-HA leu2-3 trp1-1 his3-11* | This study |
| JPY064 | *MATalpha Δclb2::LEU2 URA3::GAL-CDC6-T39A-T368A-HA leu2-3 trp1-1 his3-11* | This study |
| JPY074 | *MATa CDC6-9MYC::TRP1 cdc4-1 ade2-1 his3-11 leu2-3 trp1-1 ura3-1* | This study |
| JPY217 | *MATa URA3::CDC6-T7A-T23A-T39A-S43A-T368A-S372A-13MYC cdc4-1 ADE2 his3-11 leu2-3 trp1-1* | This study |
| JPY222 | *MATalpha URA3::CDC6-T23A-T39A-S43A-T368A-S372A-13MYC cdc4-1 ade2-1 his3-11 leu2-3 trp1-1* | This study |
| JPY117 | *MATa bar 1 CDC6-9MYC::TRP1 cdc14-3 ade2-1 leu2-3 trp1-1 ura3-1 his3-11* | This study |
| JPY129 | *MATa bar 1 CDC6-9MYC::TRP1 cdc15-1 ade2-1 leu2-3 trp1-1 ura3-1 his3-11* | This study |
